# Childhood Adversity and Anxiety, Depression, and Suicidal Behaviors Among African‐Origin Emerging Adults in the United States: Findings From an Online Panel

**DOI:** 10.1002/jcop.70155

**Published:** 2026-10-07

**Authors:** William Byansi, Eric Williamson, JaNiene E. Peoples, Robert O. Motley

**Affiliations:** ^1^ Boston College School of Social Work Newton Massachusetts USA; ^2^ Boston College Lynch School of Education and Human Development Newton Massachusetts USA; ^3^ Florida State University College of Social Work Tallahassee Florida USA

**Keywords:** adverse childhood experiences, African‐origin emerging adults, anxiety, depression, mental health, suicidal ideation, suicide attempt

## Abstract

This study examined associations between adverse childhood experiences (ACEs) and anxiety symptoms, depressive symptoms, lifetime suicidal ideation, and lifetime suicide attempt among African‐origin emerging adults in the United States. Data were drawn from a cross‐sectional, nonprobability online panel of 537 participants aged 18–29, comprising foreign‐born first‐generation African immigrants and U.S.‐born second‐generation participants with African‐born parents. We assessed ACEs using an expanded cumulative index that included conventional household adversities and four community‐level adversities. We used separate multivariable linear and logistic regression models to examine associations between ACEs and each mental health outcome. We also ran generation‐stratified models and formal tests of ACEs × generation interactions. Higher ACE scores were associated with greater anxiety, depressive symptom severity, and higher odds of lifetime suicidal ideation and lifetime suicide attempt. Associations were statistically significant within both generational groups. Generation significantly moderated the associations between ACEs and suicidal ideation and suicide attempt, with stronger associations among first‐generation participants. Generation did not significantly moderate the associations of ACEs with anxiety or depression. Second‐generation participants had higher overall odds of suicidal ideation than first‐generation participants. ACEs were consistently associated with adverse mental health outcomes among African‐origin emerging adults in this U.S.‐based online panel. The findings support culturally responsive, trauma‐informed assessment and intervention while underscoring the need for longitudinal research examining how developmental, migration‐related, and sociocultural contexts may contribute to these associations.

## 1 Introduction

Childhood adversity, commonly measured using Adverse Childhood Experiences (ACEs), is a significant public health concern associated with lifelong mental health consequences. Globally, approximately 16.1% of adults report experiencing four or more ACEs (Madigan et al. 2023). Among African immigrants in the United States (U.S.), 43% reported four or more ACEs, with emotional abuse, neglect, parental loss, and household substance use among the most frequently reported adversities (Saasa et al. 2026). ACEs have consistently been associated with anxiety, depression, and suicidal behaviors across diverse populations (Byansi et al. 2025; Byansi et al. 2023; Felitti et al. 2019; Hatcher et al. 2019; Kogan et al. 2025; Petruccelli et al. 2019). African immigrant populations also experience a substantial burden of mental health conditions, with pooled prevalence estimates of 34.6% for anxiety, 33.2% for depression, and 37.9% for post‐traumatic stress disorder (James et al. 2022). Previous research suggests that these mental health challenges may be influenced by childhood adversity together with broader social and cultural stressors, including discrimination, acculturative stress, socioeconomic disadvantage, and limited access to culturally responsive mental health services (Mesa‐Vieira et al. 2022; Motley RO et al. (2024); Tutlam et al. (2024); Kogan et al. 2025; Saasa et al. 2026). However, the extent to which these contextual factors contribute to mental health outcomes among African‐origin emerging adults remains poorly understood.

This study is guided by the cumulative risk and ecological systems perspectives, which propose that exposure to multiple adversities across family and community contexts may have additive effects on psychological well‐being (Bronfenbrenner 1979; Evans et al. 2013). Consistent with these perspectives, we conceptualize ACEs as cumulative exposures rather than isolated events. Because the present study does not measure the timing, duration, or migration context of individual adversities, it examines cumulative childhood adversity rather than developmental or migration‐stage effects.

Emerging adulthood (ages 18–29) is a developmental period marked by identity exploration and heightened stress as young people navigate major transitions in education, work, relationships, and sociocultural roles (Arnett 2000; Hope et al. 2015). For African‐origin emerging adults in the United States, these developmental transitions may coincide with additional sociocultural stressors, including racial discrimination, acculturative stress, bicultural identity negotiation, intergenerational cultural conflict, financial strain, and stigma surrounding mental health and help‐seeking (Mesa‐Vieira et al. 2022; Tutlam et al. 2024). Significantly, early adversity exerts amplified effects during emerging adulthood, as individuals with high ACE exposure demonstrate elevated risk for anxiety, depression, and suicidality (Hughes et al. 2017; Kogan et al. 2025; Mesa‐Vieira et al. 2022). These experiences may differ for first‐generation immigrants and U.S.‐born second‐generation individuals with African‐born parents. Although these contextual factors were not measured in the present study, they provide an important backdrop for understanding mental health during emerging adulthood.

The African immigrant population in the United States has expanded rapidly, with more than two million African immigrants residing in the country as of 2019, a 52% increase since 2010, driven by arrivals from Nigeria, Ethiopia, Ghana, Kenya, Somalia, and other countries, many of whom are youth and emerging adults (Batalova and Lorenzi 2019; Tamir 2022). Despite this growth, African‐origin populations remain underrepresented in mental health research and are often combined with broader Black populations, obscuring important differences in migration histories, cultural experiences, and mental health risks (Kosidou et al. 2012; Nyanamba et al. 2024). Furthermore, substantial heterogeneity exists across African‐origin populations with respect to country of origin, migration pathways, sociopolitical histories, and cultural backgrounds, factors that remain largely unexplored in existing research (Motley RO et al. (2024).

Research examining ACEs among African‐origin emerging adults remains limited. Existing studies have linked ACEs to emotional distress and social exclusion (Saasa et al. 2026), but few have examined associations with anxiety, depression, suicidal ideation, and suicide attempts within the same study population. Anxiety and depression were selected because they are among the most common mental health outcomes associated with childhood adversity and are highly prevalent among African migrant populations (James et al. 2022). Suicidal ideation and suicide attempts were examined separately because they represent distinct suicide‐related outcomes with different clinical significance and risk profiles. Understanding how ACEs relate to each outcome may provide a more comprehensive picture of mental health among African‐origin emerging adults. The study extends cumulative‐risk research by examining whether an expanded adversity index spanning household and community contexts is associated with multiple, separately modeled mental health outcomes in an understudied African‐origin population. It also tests whether immigrant generation modifies these associations, while recognizing that generational status is an imperfect proxy for the cultural, developmental, and migration‐related processes that may underlie group differences.

The present study examined associations between cumulative ACEs and anxiety, depression, suicidal ideation, and suicide attempts among African‐origin emerging adults aged 18–29 recruited through a U.S.‐based online panel. The sample included foreign‐born first‐generation African immigrants and U.S.‐born second‐generation emerging adults with African‐born parents. Separate regression models were estimated for each mental health outcome, followed by formal interaction analyses to evaluate whether associations differed by immigrant generation. By examining both conventional household‐ and community‐level adversities within an expanded ACE framework, this study extends existing evidence on childhood adversity among an understudied population and informs future longitudinal research on the developmental and sociocultural factors that may shape these associations.

## 2 Materials and Methods

### 2.1 Procedures and Participants

A University institutional review board approved all study procedures. A total of 1,378 potential participants were recruited through Dynata, a global online research platform that uses a double opt‐in panel and proprietary sampling methodology to reach diverse populations across the United States (Dynata 2024). Dynata maintains a panel of more than 60 million consumers globally and adheres to industry standards for data quality and ethical research, including European Society for Opinion and Marketing Research guidelines (Dynata 2024). Each potential participant received an electronic invitation containing a hyperlink to the survey, an explanation of the study's purpose, and an informed consent notification. Participant consent was implied when respondents clicked on the survey hyperlink. Each participant received Dynata's standard incentive for completing a 30‐min survey, which is provided in the panel's local currency and can be redeemed for cash, gift cards, or charitable donations.

Data were collected from May through July 2025 as part of a cross‐sectional nonprobability online panel survey. A total of 537 African‐origin emerging adults aged 18–29 residing in the United States met eligibility criteria and completed the online survey. The sample included foreign‐born first‐generation African immigrants and U.S.‐born second‐generation participants with African‐born parents. No quotas were applied during recruitment, and all eligible respondents within the Dynata panel who met screening criteria were invited to participate. Because this was a nonprobability sample without quota controls, findings may not be generalizable to the broader African‐origin emerging adult population in the United States. Results should be interpreted as exploratory and hypothesis‐generating. However, the use of a nationally accessible Dynata panel platform improved geographic and demographic reach.

### 2.2 Measures

#### 2.2.1 Demographic Covariates

Participants self‐reported demographic information, including sex assigned at birth, age, immigration status, years in the United States, geographic region, education attainment, college enrollment status, employment status, and personal income. Sex was coded as a binary variable (male or female). Age and years in the United States were measured continuously. Immigration status was assessed by asking participants whether they identified as first‐generation (born outside the United States) or second‐generation (born in the United States with parents born in another country). Geographic region was categorized based on zip code: Northeast, Midwest, South, and West. Education level was grouped into four categories: less than high school, high school graduate (including GED), some college or associate degree, and college graduate (bachelor's degree or higher). College enrollment was coded as a binary variable (yes or no). Employment status during the past 12 months was categorized as unemployed, part‐time, or full‐time. Personal income was assessed using four brackets: less than $10,000; $10,000 to $39,999; $40,000 to $69,999; and $70,000 or more. These covariates were selected based on prior research documenting their associations with mental health in immigrant populations (Mesa‐Vieira et al. 2022; Nyanamba et al. 2024).

#### 2.2.2 Adverse Childhood Experiences (ACEs)

Childhood adversity was measured using the Expanded ACEs framework described by Cronholm et al. (Cronholm et al. 2015), which incorporates both household and community‐level adversities. The 20‐item instrument includes Conventional ACEs (emotional, physical, and sexual abuse; emotional and physical neglect; and household dysfunction due to substance use, mental illness, domestic violence, or incarceration; (Felitti et al. 1998) as well as expanded ACEs reflecting community‐level stressors (unsafe neighborhood conditions, low neighborhood cohesion, peer bullying, and foster‐care involvement). Participants reported whether each adversity had occurred during childhood, consistent with the Expanded ACE framework; however, the instrument does not capture the age at which each event occurred, its duration, or whether it occurred before or after migration to the United States. Each category is scored dichotomously (0 = no, 1 = yes), and the total ACEs score is calculated by summing endorsed items, with a score ranging from 0 to 20. Higher scores indicate greater cumulative exposure to childhood adversity. We used the expanded ACEs measure because it captures community‐level adversities, neighborhood safety, neighborhood cohesion, peer victimization, and foster‐care involvement that extend beyond the household‐focused content of the traditional 10‐item ACE scale and are relevant to children's broader developmental environments (Cronholm et al. 2015). Consistent with cumulative risk theory, the total ACE score was analyzed as a cumulative exposure index rather than a reflective psychometric scale; therefore, the score represents the accumulation of adversities rather than a single underlying construct. The measure does not include a racial‐discrimination item.

#### 2.2.3 Depression

Depressive symptoms were measured using the Patient Health Questionnaire‐9 (PHQ‐9), a 9‐item self‐report scale assessing the frequency of depression symptoms over the past 2 weeks (Kroenke et al. 2001). Items correspond to DSM criteria for major depressive disorder and are rated on a 4‐point scale from 0 (not at all) to 3 (nearly every day). Total scores range from 0 to 27, with higher scores indicating greater symptom severity. The PHQ‐9 has demonstrated strong psychometric properties and is widely used in both clinical and research settings (Kroenke and Spitzer 2002). The Cronbach's alpha for the current sample was 0.91.

#### 2.2.4 Generalized Anxiety

Anxiety symptoms were measured using the Generalized Anxiety Disorder‐7 (GAD‐7; (Spitzer et al. 2006), a 7‐item self‐report scale designed to assess generalized anxiety disorder symptoms. Items are rated on a 4‐point scale from 0 (not at all) to 3 (nearly every day) based on symptom frequency over the past 2 weeks. Total scores range from 0 to 21, with higher values reflecting greater anxiety severity. The measure showed strong psychometric properties in the present study (*α* = 0.92).

#### 2.2.5 Suicidal Behavior

Lifetime suicidal behavior was assessed using questions from the baseline National Comorbidity Survey (Kessler et al. 1999). Suicidal ideation was defined as having ever seriously thought about or made a plan for suicide, whereas suicide attempt referred to ever attempting suicide. Both outcomes were analyzed separately as binary variables (0 = no, 1 = yes).

### 2.3 Statistical Analysis

All statistical analyses were conducted using R statistical software version 4.4.0 (R Core Team 2024), with significance set at *α* = 0.05. Descriptive statistics were calculated for all study variables, and chi‐square tests and independent‐samples t‐tests examined differences between first‐ and second‐generation immigrants. To examine the relationship between ACEs and each mental health outcome, we conducted separate multiple linear regression analyses for anxiety and depression and separate binary logistic regression analyses for suicidal ideation and suicide attempt while controlling for demographic covariates. We first examined the main effects of ACEs on each mental health outcome in the overall sample, with immigration status included as a covariate. Then, we conducted stratified analyses separately for first‐ and second‐generation immigrants to examine whether ACEs effects differed by generational status. Primary analyses used complete‐case (listwise) deletion, and multiple imputation was conducted as a sensitivity analysis to assess the robustness of the findings.

We conducted several supplementary and sensitivity analyses to evaluate the robustness and dimensionality of the ACE exposure. Because the expanded ACE index is a cumulative‐risk count rather than a reflective scale, we examined item‐level endorsement rates and, following Cronholm et al. (2015), two conceptual domains: conventional/household adversities (items 1–16) and community/expanded adversities (items 17–20). We entered the two domain sub‐scores simultaneously in each outcome model to determine whether associations were attributable to a particular domain, and we report internal‐consistency coefficients (KR‐20) for descriptive purposes while noting that internal consistency is not an appropriate psychometric criterion for a cumulative‐exposure index. We assessed the linearity of the ACE–outcome relationship by adding a quadratic ACE term to each model. To test directly whether ACE associations differed by immigrant generation—rather than inferring this from separate stratified models—we estimated models that included an ACEs × Generation interaction term. For the suicide‐attempt outcome, where events were sparse (particularly among first‐generation participants), we re‐estimated the models using Firth penalized logistic regression (Firth 1993) to reduce small‐sample and separation bias. We also re‐estimated the suicidality models adjusting for concurrent depression (PHQ‐9) and anxiety (GAD‐7) symptoms to assess whether ACE associations were independent of current distress. Finally, although missing data were minimal, we conducted a multiple‐imputation sensitivity analysis (20 imputations; van Buuren and Groothuis‐Oudshoorn 2011) and compared the pooled estimates with the complete‐case results.

## 3 Results

### 3.1 Sample Characteristics by Immigrant Generation

The final analytic sample included 537 African‐origin emerging adults, of whom 23.3% (*n* = 125) were first‐generation, and 76.7% (*n* = 412) were second‐generation immigrants (see Table 1). The average age was 24.7 years (SD = 3.0), and sex distribution did not differ by immigrant generation. First‐generation participants had lived significantly fewer years in the U.S. (M = 12.2, SD = 7.89) than second‐generation participants (*M* = 22.3, SD = 5.83; *t* = −13.262, *p* < 0.001). Educational attainment, college enrollment, employment status, and income levels were similar across groups.

**Table 1:** Bivariate relationships between immigrant status and study variables.

Variable	First generation	Second generation	Test
	(*n* = 125)	(*n* = 412)	χ2 or *t‐test*
Age, M (SD)	24.6 (3.20)	24.7 (2.96)	−0.415 (df = 193)
Assigned sex at birth, *n* (%)			0.100 (df = 1)
Male	62 (49.6)	211 (51.2)	
Female	63 (50.4)	201 (48.8)	
Years lived in the U.S., M (SD)	12.2 (7.89)	22.3 (5.83)	−13.262 (df = 167)***
Region, *n* (%)			2.787 (df = 3)
Northeast	23 (20)	70 (18.2)	
Midwest	27 (23.5)	74 (19.2)	
South	45 (39.1)	184 (47.8)	
West	20 (17.4)	57 (14.8)	
Educational attainment, *n* (%)			*p* = 0.242^a^
Less than high school	5 (4)	5 (1.2)	
High school graduate	40 (32)	132 (32)	
Some college	20 (16)	78 (18.9)	
College graduate	60 (48)	197 (47.8)	
Currently enrolled in college, *n* (%)			0.00 (df = 1)
Yes	42 (33.6)	138 (33.5)	
No	83 (66.4)	274 (66.5)	
Employment, *n* (%)			2.667 (df = 2)
Unemployed	68 (54.4)	257 (62.4)	
Part time	16 (12.8)	47 (11.4)	
Full time	41 (32.8)	108 (26.2)	
Income, *n* (%)			3.747 (df = 3)
Less than $10,000	20 (16)	48 (11.7)	
$10,000–$39,999	39 (31.2)	130 (31.6)	
$40,000–$69,999	34 (27.2)	97 (23.5)	
$70,000 or more	32 (25.6)	137 (33.3)	
Number of ACEs, *n* (%)			4.957 (df = 3)
0	1 (0.8)	0 (0.0)	
1–5	42 (33.9)	114 (28.1)	
6–10	65 (52.4)	236 (58.1)	
11 or more	16 (12.9)	56 (13.8)	
Generalized anxiety, M (SD)	7.95 (6.0)	8.50 (6.05)	−0.893 (df = 206)
Depression, M (SD)	8.67 (6.70)	9.82 (7.36)	−1.637 (df = 223)
Suicidal ideation, *n* (%)			
Yes	31 (24.8)	156 (37.9)	6.647 (df = 1)*
No	94 (75.2)	256 (62.1)	
Suicide attempt, *n* (%)			
Yes	17 (13.6)	60 (14.6)	0.015 (df = 1)
No	108 (86.4)	352 (85.4)	

*Note: N* = 537 (1st generation *n* = 125; 2nd generation *n* = 412). Continuous variables are reported as *M* (SD) and compared using Welch's independent‐samples *t* tests; categorical variables are reported as *n* (%) and compared using Pearson χ^2^ tests, with degrees of freedom shown in parentheses. Percentages are based on the valid *n* for each variable: region was missing for 37 participants (first generation *n* = 10; second generation *n* = 27) and total ACE score for 7 participants (first generation *n* = 1; second generation *n* = 6); all other variables were complete. The college‐enrollment χ^2^ of 0.00 reflects nearly identical enrollment rates across generations. ACEs = adverse childhood experiences.

^a^ Fisher‐Freeman‐Halton exact test; an exact *p‐value* is reported in place of χ^2^ because expected cell counts were less than 5.

* *p* < 0.05

*** *p* < 0.001.

The distribution of ACEs did not differ significantly between groups (χ^2^ = 4.96, df = 3, *p* = 0.18), with 33.9% of first‐generation participants reporting 1–5 ACEs compared to 28.1% of second‐generation participants. One first‐generation participant reported zero ACEs and was retained in all analyses; all others endorsed at least one. Mean generalized anxiety scores were 7.95 (SD = 6.0) for first‐generation and 8.50 (SD = 6.05) for second‐generation participants. Mean depression scores were 8.67 (SD = 6.70) for first‐generation and 9.82 (SD = 7.36) for second‐generation participants. Lifetime suicidal ideation prevalence differed significantly (χ^2^ = 6.647, *p* = 0.02), with second‐generation participants reporting higher rates (37.9%) compared to first‐generation participants (24.8%). Distinguishing the components of the ideation measure, 24.8% of first‐generation and 36.9% of second‐generation participants reported suicidal thoughts, and 12.0% and 16.7%, respectively, reported having made a suicide plan. Suicide attempt prevalence was similar between groups.

### 3.2 ACES and Anxiety

Linear regression analyses revealed a significant positive association between ACEs and anxiety symptom severity in the overall sample (B = 0.840, 95% CI [0.661, 1.019], *p* < 0.001; see Table 2). This pattern held in generation‐stratified analyses, with significant effects observed among both first‐generation (B = 1.143, 95% CI [0.759, 1.526], *p* < 0.001) and second‐generation (B = 0.754, 95% CI [0.546, 0.963], *p* < 0.001) immigrant participants. Among second‐generation participants, income between $10,000 and $39,999 (vs. less than $10,000) was associated with higher anxiety symptom (B = 2.240, 95% CI [0.144, 4.335]). No other demographic variables emerged as significant predictors in either the overall or stratified models.

**Table 2:** Linear regression results: ACEs predicting anxiety symptom severity.

Variable	Overall B [95% CI]	First generation B [95% CI]	Second generation B [95% CI]
ACEs (Total)	0.840 [0.661, 1.019]***	1.143 [0.759, 1.526]***	0.754 [0.546, 0.963]***
Age	−0.143 [−0.333, 0.046]	−0.001 [−0.386, 0.384]	−0.212 [−0.439, 0.015]
Years in U.S.	−0.009 [−0.093, 0.075]	0.014 [−0.120, 0.149]	−0.028 [−0.138, 0.082]
Sex: Female	−0.084 [−1.098, 0.930]	2.094 [−0.127, 4.315]	−0.837 [−2.004, 0.331]
Midwest	0.188 [−1.420, 1.796]	−0.214 [−3.539, 3.110]	−0.188 [−2.074, 1.699]
South	−1.026 [−2.400, 0.347]	−2.167 [−5.053, 0.719]	−1.090 [−2.686, 0.506]
West	−0.283 [−1.995, 1.429]	−0.539 [−3.956, 2.878]	−0.393 [−2.412, 1.626]
HS Graduate	−1.115 [−4.991, 2.761]	0.725 [−4.744, 6.193]	−4.169 [−9.925, 1.587]
Some College	0.256 [−3.731, 4.243]	1.984 [−3.920, 7.889]	−2.603 [−8.422, 3.217]
College Graduate	−1.068 [−5.020, 2.883]	0.264 [−5.527, 6.055]	−3.893 [−9.685, 1.900]
College Enrolled: No	−0.737 [−1.859, 0.385]	‐0.359 [−2.656, 1.939]	−0.809 [−2.110, 0.492]
Part‐time	−0.602 [−2.430, 1.225]	−0.167 [−3.801, 3.468]	−0.576 [−2.712, 1.559]
Full‐time	0.152 [−1.050, 1.353]	1.257 [−1.185, 3.700]	−0.011 [−1.422, 1.400]
$10k‐$39,999	1.121 [−0.622, 2.865]	−2.024 [−5.327, 1.280]	2.240 [0.144, 4.335]*
$40k‐$69,999	1.000 [−0.944, 2.945]	−2.204 [−5.733, 1.325]	2.217 [−0.142, 4.577]
$70k or more	0.479 [−1.474, 2.432]	−0.759 [−4.490, 2.972]	1.108 [−1.235, 3.450]
Immigrant Status: 2nd Gen	0.597 [−0.829, 2.022]	—	—
*R* ^2^	0.189	0.340	0.184
Adjusted *R* ^2^	0.160	0.231	0.148
*N*	493	114	379

*Note: N* = 493 (1st generation *n* = 114; 2nd generation *n* = 379). Analytic samples are smaller than the descriptive sample reported in Table 1 (*N* = 537) because models were estimated with listwise deletion on region (37 missing) and total ACE score (7 missing). *B* = unstandardized regression coefficient; CI = confidence interval; HS = high school; ACEs = adverse childhood experiences. Reference categories are male (sex), Northeast (region), less than high school (educational attainment), currently enrolled in college (college enrollment), unemployed (employment), less than $10,000 (income), and 1st generation (immigrant generation). Immigrant generation is entered as a covariate only in the overall model; the generation‐specific models are estimated within each subgroup and therefore do not include this term. ACEs (Total) is the count of endorsed adverse childhood experiences, entered as a continuous predictor.

* *p* < 0.05

*** *p* < 0.001.

### 3.3 ACES and Depression

Linear regression analyses indicated a significant positive association between ACEs and depressive symptom severity in the overall sample (B = 0.919, 95% CI [0.702, 1.137], *p* < 0.001; see Table 3). Southern U.S. residence was also significantly associated with lower depression scores (B = −1.902, 95% CI [−3.572, −0.233], *p* < 0.05). Generation‐stratified models showed that ACEs significantly predicted higher depression among both first‐generation (B = 1.277, 95% CI [0.851, 1.704], *p* < 0.001) and second‐generation (B = 0.802, 95% CI [0.545, 1.058], *p* < 0.001) participants. Within the second‐generation subsample, Southern residence remained a significant predictor of lower depressive symptoms (B = −2.634, 95% CI [−4.601, −0.667], *p* < 0.01), and income between $40,000 and $69,999 was associated with higher depression scores (B = 3.301, 95% CI [0.393, 6.209], *p* < 0.05). Female sex was additionally associated with lower depression (B = −1.542, 95% CI [−2.981, −0.103]), and income between $10,000 and $39,999 with higher depression (B = 2.739, 95% CI [0.157, 5.322]), among second‐generation participants. No other demographic variables were significantly associated with depressive symptom severity in either the overall or stratified models.

**Table 3:** Linear regression results: ACEs predicting depression symptom severity.

Variable	Overall B [95% CI]	First generation B [95% CI]	Second generation B [95% CI]
ACEs (Total)	0.919 [0.702, 1.137]***	1.277 [0.851, 1.704]***	0.802 [0.545, 1.058]***
Age	−0.101 [−0.332, 0.129]	0.011 [−0.417, 0.440]	−0.183 [−0.462, 0.096]
Years in U.S.	−0.022 [−0.124, 0.080]	−0.017 [−0.167, 0.132]	−0.033 [−0.169, 0.102]
Sex: Female	−0.536 [−1.768, 0.697]	1.826 [−0.643, 4.295]	−1.542 [−2.981, −0.103]*
Midwest	−0.007 [−1.961, 1.948]	−0.563 [−4.259, 3.133]	−0.646 [−2.971, 1.679]
South	−1.902 [−3.572, −0.233]*	−1.599 [−4.808, 1.609]	−2.634 [−4.601, −0.667]
West	0.017 [‐2.064, 2.098]	0.409 [−3.389, 4.208]	−0.528 [−3.017, 1.960]
HS Graduate	−1.120 [−5.831, 3.591]	3.423 [−2.656, 9.502]	−6.192 [−13.287, 0.904]
Some College	−0.460 [−5.306, 4.386]	2.219 [−4.344, 8.782]	−4.781 [−11.954, 2.393]
College Graduate	−2.103 [−6.906, 2.700]	1.469 [−4.968, 7.906]	−6.783 [−13.922, 0.357]
College enrolled: No	−0.810 [−2.174, 0.554]	0.623 [−1.931, 3.177]	−1.221 [−2.824, 0.383]
Part‐time	−0.292 [−2.514, 1.930]	0.395 [−3.645, 4.435]	−0.356 [−2.988, 2.276]
Full‐time	−0.057 [−1.517, 1.404]	1.588 [−1.127, 4.303]	−0.393 [−2.132, 1.346]
$10,000–$39,999	1.606 [−0.513, 3.725]	−2.157 [−5.829, 1.515]	2.739 [0.157, 5.322]*
$40,000–$69,999	1.937 [−0.427, 4.300]	−2.033 [−5.956, 1.889]	3.301 [0.393, 6.209]*
$70k or more	1.200 [−1.174, 3.574]	−0.414 [−4.561, 3.734]	1.720 [−1.167, 4.607]
Immigrant status: Second generation	1.415 [−0.318, 3.148]	—	—
*R* ^2^	0.171	0.346	0.172
Adjusted *R* ^2^	0.141	0.238	0.136
*N*	493	114	379

*Note: N* = 493 (1st generation *n* = 114; 2nd generation *n* = 379). Analytic samples are smaller than the descriptive sample reported in Table 1 (*N* = 537) because models were estimated with listwise deletion on region (37 missing) and total ACE score (7 missing). *B* = unstandardized regression coefficient; CI = confidence interval; HS = high school; ACEs = adverse childhood experiences. Reference categories are male (sex), Northeast (region), less than high school (educational attainment), currently enrolled in college (college enrollment), unemployed (employment), less than $10,000 (income), and First generation (immigrant generation). Immigrant generation is entered as a covariate only in the overall model; the generation‐specific models are estimated within each subgroup and therefore do not include this term. ACEs (Total) is the count of endorsed adverse childhood experiences, entered as a continuous predictor.

* *p* < 0.05

*** *p* < 0.001.

### 3.4 ACEs and Suicidal Ideation

Logistic regression analyses showed that ACEs were significantly associated with increased odds of lifetime suicidal ideation in the overall sample (OR = 1.331, 95% CI [1.232, 1.444], *p* < 0.001; see Table 4). Second‐generation immigrant status was associated with significantly higher odds of suicidal ideation (OR = 2.145, 95% CI [1.168, 4.035], *p* < 0.05). In comparison, full‐time employment was associated with significantly lower odds (OR = 0.572, 95% CI [0.339, 0.951], *p* < 0.05). In generation‐stratified models, ACEs remained significant predictors for both first‐generation (OR = 1.775, 95% CI [1.398, 2.396], *p* < 0.001) and second‐generation (OR = 1.263, 95% CI [1.161, 1.381], *p* < 0.001) participants. Other demographic variables were not significantly associated with suicidal ideation in the overall or stratified models.

**Table 4:** Logistic regression results: ACEs predicting suicidal ideation.

Variable	Overall OR [95% CI]	First generation OR [95% CI]	Second generation OR [95% CI]
ACEs (Total)	1.331 [1.232, 1.444]***	1.775 [1.398, 2.396]***	1.263 [1.161, 1.381]***
Age	1.009 [0.933, 1.091]	1.137 [0.909, 1.443]	0.978 [0.894, 1.069]
Years in U.S.	0.990 [0.956, 1.025]	0.970 [0.895, 1.046]	0.997 [0.955, 1.042]
Sex: Female	1.452 [0.956, 2.211]	0.947 [0.273, 3.176]	1.446 [0.913, 2.301]
Midwest	0.833 [0.427, 1.620]	2.597 [0.361, 22.477]	0.713 [0.340, 1.486]
South	0.717 [0.408, 1.266]	1.861 [0.355, 11.869]	0.582 [0.311, 1.088]
West	0.956 [0.469, 1.938]	1.673 [0.199, 15.499]	0.840 [0.379, 1.844]
HS Graduate	0.832 [0.172, 4.804]	0.208 [0.013, 5.855]	0.962 [0.102, 9.051]
Some College	1.908 [0.378, 11.359]	0.408 [0.020, 13.535]	2.599 [0.267, 25.066]
College Graduate	0.875 [0.174, 5.159]	0.192 [0.010, 5.984]	1.095 [0.114, 10.415]
College Enrolled: No	1.078 [0.680, 1.717]	0.889 [0.261, 3.130]	1.097 [0.657, 1.841]
Part‐time	1.282 [0.608, 2.694]	0.745 [0.086, 5.579]	1.440 [0.628, 3.303]
Full‐time	0.572 [0.339, 0.951]*	0.370 [0.074, 1.624]	0.623 [0.349, 1.092]
$10k‐$39,999	1.050 [0.503, 2.229]	3.575 [0.388, 54.948]	0.895 [0.391, 2.070]
$40k‐$69,999	1.066 [0.467, 2.474]	1.979 [0.190, 29.998]	0.931 [0.364, 2.406]
$70k or more	0.997 [0.437, 2.313]	3.081 [0.268, 53.378]	0.822 [0.324, 2.101]
Immigrant Status: 2nd Gen	2.145 [1.168, 4.035]*	—	—
*N*	493	114	379

*Note: N* = 493 (First generation *n* = 114; Second generation *n* = 379). Analytic samples are smaller than the descriptive sample reported in Table 1 (*N* = 537) because models were estimated with listwise deletion on region (37 missing) and total ACE score (7 missing). *OR* = odds ratio; CI = confidence interval; HS = high school; ACEs = adverse childhood experiences. Odds ratios are from binary logistic regression models predicting endorsement of lifetime suicidal ideation (reference category = no ideation). Reference categories are male (sex), Northeast (region), less than high school (educational attainment), currently enrolled in college (college enrollment), unemployed (employment), less than $10,000 (income), and 1st generation (immigrant generation). Immigrant generation is entered as a covariate only in the overall model; the generation‐specific models are estimated within each subgroup and therefore do not include this term. ACEs (Total) is the count of endorsed adverse childhood experiences, entered as a continuous predictor.

* *p* < 0.05

*** *p* < 0.001.

### 3.5 ACES and Suicide Attempts

Logistic regression analyses indicated that ACEs were significantly associated with increased odds of lifetime suicide attempts in the overall sample (OR = 1.310, 95% CI [1.192, 1.447], *p* < 0.001; see Table 5). In generation‐stratified models, ACEs remained significant predictors among both first‐generation (OR = 1.762, 95% CI [1.314, 2.603], *p* < 0.001) and second‐generation (OR = 1.261, 95% CI [1.129, 1.412], *p* < 0.001) immigrants. Among first‐generation participants, older age predicted greater odds of a suicide attempt (OR = 1.491, 95% CI [1.093, 2.220], *p* < 0.05); however, given the small number of attempt events in this subgroup, this estimate should be interpreted with caution, and a penalized (Firth) model did not retain it (see sensitivity analyses). Among second‐generation participants, older age (OR = 0.841, 95% CI [0.740, 0.950]) and Southern residence (OR = 0.377, 95% CI [0.164, 0.876]) were associated with lower odds of a suicide attempt. No other demographic variables were significantly associated with suicide attempt risk in the overall or stratified models.

**Table 5:** Logistic regression results: ACEs predicting suicide attempt.

Variable	Overall OR [95% CI]	First generation OR [95% CI]	Second generation OR [95% CI]
ACEs (Total)	1.310 [1.192, 1.447]***	1.762 [1.314, 2.603]***	1.261 [1.129, 1.412]***
Age	0.935 [0.844, 1.035]	1.491 [1.093, 2.220]*	0.841 [0.740, 0.950]**
Years in U.S.	0.983 [0.939, 1.031]	0.990 [0.888, 1.099]	0.997 [0.937, 1.073]
Sex: Female	1.493 [0.858, 2.625]	3.847 [0.762, 26.303]	1.179 [0.623, 2.245]
Midwest	0.697 [0.296, 1.632]	3.117 [0.154, 82.464]	0.554 [0.212, 1.421]
South	0.568 [0.274, 1.205]	5.411 [0.383, 126.048]	0.377 [0.164, 0.876]*
West	0.646 [0.248, 1.626]	10.690 [0.558, 353.706]	0.375 [0.119, 1.099]
HS Graduate	0.615 [0.120, 4.664]	0.097 [0.002, 4.424]	0.481 [0.049, 10.866]
Some College	1.378 [0.258, 10.743]	0.111 [0.002, 7.925]	1.667 [0.173, 37.505]
College Graduate	0.498 [0.091, 3.930]	0.012 [0.000, 0.792]*	0.519 [0.053, 11.755]
College enrolled: No	1.556 [0.839, 2.978]	0.722 [0.129, 3.895]	1.892 [0.923, 4.064]
Part‐time	1.167 [0.454, 2.860]	0.475 [0.019, 7.486]	1.552 [0.517, 4.397]
Full‐time	0.632 [0.310, 1.236]	0.824 [0.130, 4.869]	0.696 [0.306, 1.501]
$10,000–$39,999	1.329 [0.540, 3.462]	3.053 [0.233, 76.719]	1.539 [0.528, 4.824]
$40,000–$69,999	0.981 [0.330, 2.994]	0.553 [0.022, 14.074]	1.512 [0.416, 5.752]
$70,000 or more	1.265 [0.443, 3.767]	1.166 [0.050, 34.649]	1.528 [0.448, 5.520]
Immigrant Status: Second generation	1.135 [0.523, 2.551]	—	—
*N*	493	114	379

*Note: N* = 493 (First generation *n* = 114; Second generation *n* = 379). Analytic samples are smaller than the descriptive sample reported in Table 1 (*N* = 537) because models were estimated with listwise deletion on region (37 missing) and total ACE score (7 missing). *OR* = odds ratio; CI = confidence interval; HS = high school; ACEs = adverse childhood experiences. Odds ratios are from binary logistic regression models predicting endorsement of a lifetime suicide attempt (reference category = no attempt). Reference categories are male (sex), Northeast (region), less than high school (educational attainment), currently enrolled in college (college enrollment), unemployed (employment), less than $10,000 (income), and First generation (immigrant generation). Immigrant generation is entered as a covariate only in the overall model; the generation‐specific models are estimated within each subgroup and therefore do not include this term. ACEs (Total) is the count of endorsed adverse childhood experiences, entered as a continuous predictor. The 1st‐generation College Graduate estimate reflects near‐complete separation (few events) and should not be interpreted.

* *p* < 0.05

** *p* < 0.01

*** *p* < 0.001.

### 3.6 Missing Data

Of the 537 participants, 493 (91.8%) had complete data on all analytic variables. Missingness was confined to geographic region (6.9%) and the total ACE score (1.3%); all four mental health outcomes and the remaining covariates were complete. The probability of being a complete case did not differ by immigrant generation (χ^2^ = 0.01, *p* = 0.92). A multiple‐imputation sensitivity analysis (20 imputations) produced pooled ACE estimates that were essentially identical to the complete‐case results (e.g., anxiety *B* = 0.81; depression *B* = 0.89), indicating that listwise deletion did not bias the primary findings.

### 3.7 Expanded ACE Analyses

Endorsement rates for individual adverse childhood experiences are presented in Table 6 and demonstrate variability across both conventional household and community‐level adversities. To examine whether the observed associations were driven primarily by one domain of adversity, conventional household ACEs (items 1–16) and community‐level ACEs (items 17–20) were entered simultaneously into each regression model. Both domains remained independently associated with anxiety, depression, suicidal ideation, and suicide attempt (all *p* < 0.01; Table 6), indicating that the associations were not attributable to a single domain of adversity.

**Table 6:** ACE item endorsement (%), overall and by immigrant generation.

ACE Item	Domain	Overall	First generation	Second generation
Emotional abuse (insulted or put down)	Conventional	47.9	50.4	47.1
Emotional abuse (feared harm)	Conventional	61.6	64.8	60.7
Physical abuse (pushed, grabbed, slapped)	Conventional	62.6	61.6	62.9
Physical abuse (hit, leaving marks or injury)	Conventional	74.5	79.2	73.1
Sexual abuse (contact)	Conventional	20.7	24.0	19.7
Sexual abuse (attempted or forced)	Conventional	17.9	18.4	17.7
Emotional neglect (no one made you feel special)	Conventional	3.9	7.2	2.9
Physical neglect or food insecurity	Conventional	75.6	76.0	75.5
Witnessed domestic violence (slapped, kicked)	Conventional	43.0	42.4	43.2
Witnessed domestic violence (hit, injured)	Conventional	34.3	32.0	35.0
Household alcohol problem	Conventional	35.5	31.2	36.8
Household drug problem	Conventional	23.3	19.2	24.6
Household mental illness	Conventional	36.3	28.8	38.6
Household member suicidality	Conventional	18.6	13.6	20.1
Parental separation or divorce	Conventional	37.7	32.0	39.4
Household member incarceration	Conventional	30.3	23.2	32.4
Felt unsafe in neighborhood	Community	6.0	7.2	5.6
Low neighborhood cohesion	Community	12.7	15.2	11.9
Bullied by peers	Community	67.5	67.7	67.4
Foster‐care involvement	Community	9.1	7.2	9.7

*Note:* Values are the percentage of participants endorsing each item (scored 1 = adverse). Conventional = conventional/household ACEs (items 1–16); Community = community/expanded ACEs (items 17–20). Items 7, 17, and 18 are positively worded in the source instrument and were scored so that 1 indicates the adverse response (no emotional support, felt unsafe, low cohesion). Overall *N* = 537 (First generation *n* = 125; Second generation *n* = 412); percentages use available cases for the total ACE items.

Internal consistency coefficients were low for the full expanded ACE index (KR‐20 = 0.56), the conventional ACE domain (KR‐20 = 0.53), and the community ACE domain (KR‐20 = 0.22). Because the expanded ACE measure was analyzed as a cumulative exposure index rather than a reflective psychometric scale, these coefficients are presented for descriptive purposes only. Finally, inclusion of a quadratic ACE term did not improve model fit for any outcome (e.g., anxiety, F(1, 474) = 0.57, *p* = 0.45), supporting the use of a linear specification in the primary analyses.

### 3.8 Generational Moderation

To test directly whether ACE associations differed by generation, we estimated models that included an ACEs × Generation interaction term (See Table S1 and Table S2). Interaction terms were not statistically significant for anxiety or depression, indicating insufficient evidence that ACE associations differed by immigrant generation for these outcomes. By contrast, the interaction was significant for both suicidal ideation (B = 0.71, *p* = 0.008) and suicide attempt (B = 0.73, *p* = 0.03), indicating that the association between ACEs and suicidality was significantly stronger among first‐generation participants.

### 3.9 Sensitivity Analyses for Suicidality

Two sensitivity analyses were conducted. Firth penalized logistic regression—used to address the sparse number of attempt events—reproduced the ACE association (overall OR = 1.27, 95% CI [1.16, 1.39]; first‐generation OR = 1.45, 95% CI [1.19, 1.81]; both *p* < 0.001); the first‐generation age effect observed in the standard model was not retained under the penalized model (*p* = 0.70), reinforcing a cautious interpretation of that estimate. Second, ACEs continued to predict both suicidal ideation (OR = 1.22, 95% CI [1.12, 1.32]) and suicide attempt (OR = 1.21, 95% CI [1.09, 1.34]) after adjustment for concurrent depression and anxiety symptoms, indicating that the link between childhood adversity and suicidality was not fully accounted for by current distress (See Table S3).

## 4 Discussion

This study provides one of the most comprehensive examinations of childhood adversity and mental health among African‐origin emerging adults recruited through a U.S‐based online panel. Four central findings emerged. First, greater ACEs exposure was associated with higher levels of anxiety, depression, suicidal ideation, and suicide attempts. Second, these associations were evident among both first‐ and second‐generation participants. However, formal interaction analyses indicated significant differences by immigrant generation only for suicidal ideation and suicide attempts; evidence of moderation for anxiety and depression was not statistically significant. Third, several demographic correlates varied across groups, including higher anxiety among first‐generation women, lower anxiety with older age among second‐generation participants, and lower depressive symptoms among participants living in Southern states. Finally, second‐generation participants reported higher overall levels of suicidal ideation, while the association between ACEs and suicidality differed by immigrant generation. Together, these findings demonstrate heterogeneity in the associations between childhood adversity and mental health among African‐origin emerging adults.

These findings align with broader ACEs research, which demonstrates robust associations between early adversity and internalizing symptoms across populations (Felitti et al. 2019; Hughes et al. 2017). Among African immigrants in the U.S., ACE exposure has also been associated with greater psychological distress (Saasa et al. 2026). Previous research suggests that African immigrants may encounter adversity before, during, and after migration, including conflict, poverty, family separation, discrimination, and structural exclusion (Amene et al. 2024; Fazel et al. 2012; Kosidou et al. 2012; Tutlam et al. (2024). Although these migration‐related experiences were not directly measured in the present study, they provide a broader context for considering how childhood adversity may intersect with migration and resettlement experiences. Our findings extend this literature by demonstrating associations between cumulative ACE exposure and anxiety, depression, suicidal ideation, and suicide attempts among African‐origin emerging adults.

Although ACEs were associated with mental health outcomes in both immigrant generations, significant generational differences emerged only for suicidal ideation and suicide attempts. Second‐generation participants reported higher overall levels of suicidal ideation, while interaction analyses indicated stronger associations between ACEs and both suicidal ideation and suicide attempts among first‐generation participants. Previous research has identified racial discrimination, bicultural identity challenges, and intergenerational cultural conflict as potential contributors to psychological distress and suicidality among African diaspora youth (Edelblute et al. 2024; Hope et al. 2015; Motley RO et al. 2024; Nyanamba et al. 2024; Polanco‐Roman et al. 2019). These factors could contribute to the generational patterns observed in our study, but they were not directly measured and cannot be established as explanatory mechanisms from the present data. Similarly, differences in cultural and social support, migration experiences, or the timing and context of adversity exposure may help explain why the association between ACEs and suicidality varied by immigrant generation. Future longitudinal studies should directly assess these factors to determine whether and how they account for generational differences in the effects of childhood adversity.

Several demographic findings also warrant further investigation. Female sex was associated with higher anxiety among first‐generation participants. This finding is consistent with literature describing gendered stressors among immigrant women, including caregiving demands, economic strain, and gender discrimination (Nyanamba et al. 2024). However, we did not assess these stressors, so we cannot determine their role in explaining the observed association. Older age was associated with lower anxiety among second‐generation participants. Developmental changes in coping, identity formation, or adaptation to bicultural contexts may explain this pattern, but these mechanisms require direct examination. Similarly, residence in the South was associated with lower depressive symptoms in the overall sample and among second‐generation participants. Regional differences in social networks, community resources, cultural environments, or other unmeasured characteristics could contribute to this association. Because these factors were not measured, the regional finding should be viewed as an empirical pattern requiring further investigation rather than evidence of a protective effect of residence in the South.

The association between older age and suicide attempts among first‐generation participants also requires cautious interpretation. Although this association emerged in the primary analysis, it was not retained in the Firth penalized sensitivity analysis. Given the relatively small number of suicide attempts and the instability of this estimate across analytic approaches, this finding should not be interpreted as evidence that suicide attempt risk increases with age among first‐generation African‐origin emerging adults. Replication in larger samples and longitudinal research are needed to determine whether age contributes to variation in suicidality within this population.

An important contribution of this study is the finding that both conventional household ACEs and community‐level adversities were independently associated with anxiety, depression, suicidal ideation, and suicide attempts. These findings are consistent with the Cumulative Risk Framework, which emphasizes the potential consequences of accumulated adversity across multiple domains (Evans et al. 2013) and Ecological Systems Theory, which situates development within multiple interconnected environmental contexts (Bronfenbrenner 1979). The findings therefore support examining childhood adversity beyond conventional household ACEs when studying the mental health of African‐origin emerging adults. Future research should investigate how specific household, community, migration‐related, and sociocultural adversities combine or interact to shape mental health across immigrant generations.

### 4.1 Limitations

Several limitations need to be considered when interpreting these findings. First, the study relied on a nonprobability sampling approach through the Dynata panel, which limits generalizability despite improving geographic reach. We acknowledge that the sample may underrepresent undocumented immigrants, recently arrived refugees, or individuals with limited English proficiency, who may differ in exposure to adversity and mental health outcomes. Second, the cross‐sectional design precludes causal inference and does not capture developmental changes over time. Associations should therefore be interpreted as correlational and exploratory. In addition, the expanded ACE measure assessed cumulative childhood adversity but did not capture the age at exposure, duration, frequency, or timing of adversity relative to migration. Consequently, we could not examine developmentally sensitive periods or determine whether reported adversities occurred before migration, during migration, or after resettlement. Third, all data were self‐reported, which may introduce recall bias, particularly for ACEs and suicidal behavior. Cultural stigma surrounding mental illness and suicide in many African‐origin emerging adult communities may also contribute to underreporting, especially among first‐generation adults. Fourth, while the sample included both first‐ and second‐generation emerging adults from multiple African countries, the study could not fully account for within‐group cultural heterogeneity, including regional, linguistic, and sociopolitical differences. Finally, although ACEs remained associated with suicidal ideation and suicide attempt after adjustment for depression and anxiety symptoms, the present analyses did not formally examine mediation pathways. Future longitudinal studies should investigate the mechanisms linking cumulative childhood adversity to mental health outcomes. These contextual factors likely shape both exposure to adversity and mental health trajectories in ways not captured by the current design. Finally, mental health diagnoses were not clinically assessed, limiting conclusions to symptomatology and self‐reported outcomes.

## 5 Conclusion

Despite these limitations, the study makes a significant contribution by providing one of the first U.S.‐based studies examining how childhood adversity influences mental health among African‐origin emerging adults in the U.S. The findings underscore the enduring impact of ACEs across generations and highlight how cumulative childhood adversity across multiple ecological contexts contributes to heightened risk for anxiety, depression, and suicidality. These findings advance understanding of an understudied population and address a significant health equity gap in immigrant mental health research. By documenting these patterns in a vulnerable and rapidly growing demographic, this study provides foundational evidence to guide future research and advance culturally grounded psychological assessment and intervention efforts. The results highlight the importance of attending to cumulative childhood adversity and broader sociocultural contexts when evaluating and treating African‐origin emerging adults.

Implications for practice and research are substantial. Clinicians should routinely assess ACEs among African‐origin emerging adults and consider culturally grounded expressions of distress, including somatization, stigma‐related suppression of symptoms, and faith‐centered coping. Assessment practices should incorporate culturally responsive interviewing techniques and acknowledge how migration‐related experiences, discrimination, and community dynamics shape psychological experiences. Counseling centers and community mental health agencies serving African‐origin emerging adult communities should integrate trauma‐informed care that acknowledges the impacts of racism, bicultural stress, and immigration‐related family strain.

At the policy level, these findings support the need for targeted prevention programs, expanded access to culturally competent mental health services, and investment in community organizations that provide culturally anchored support. Policies that strengthen social belonging, reduce discrimination in institutional settings, and enhance economic stability may indirectly reduce the mental health burden associated with ACEs. Future research should employ longitudinal designs to examine how ACE‐related mental health trajectories develop and should include more detailed cultural and contextual factors, such as country of origin, migration routes, and acculturation experiences, to capture within‐group diversity better. Mixed‐methods and community‐engaged approaches will be essential for deepening understanding of culturally specific pathways connecting early adversity to mental health outcomes. These steps will help build a more nuanced understanding of African‐origin emerging adult mental health and support the creation of interventions that foster resilience, healing, and equity across generations.

## Author Contributions

**William Byansi, Robert O. Motley Jr:** designed the study. **Eric Williamson:** analyzed the data. **William Byansi:** drafted the original manuscript. **JaNiene E. Peoples:** Reviewed the manuscript. All authors (**William Byansi, Eric Williamson, JaNiene E. Peoples**, and **Robert O. Motley Jr**) reviewed and revised the manuscript and approved the final article. **William Byansi** and **Robert O. Motley Jr** supervised the study.

## Ethics Statement

The study received ethical approval from the Boston College Institutional Review Board.

## Conflicts of Interest

The authors declare no conflicts of interest.

## Supporting information


Supporting File

## Data Availability

Data used in the manuscript are available from the corresponding author upon reasonable request.
