# Supplementary material for: Childhood Adversity and Anxiety, Depression, and Suicidal Behaviors Among African‐Origin Emerging Adults in the United States: Findings From an Online Panel
Source: J Community Psychol. 2026 Oct 7;54(8):e70155. doi: 10.1002/jcop.70155 (PMC13643930; doi:10.1002/jcop.70155)
Supplement: Supplementary file 1 [file JCOP-54-0-s001.docx]

**Supplementary Materials**

**Table S1**

Conventional and Community ACE Domains Entered Simultaneously in Each Outcome Model

| Outcome | Conventional ACEs (items 1-16) | Community ACEs (items 17-20) |
| --- | --- | --- |
| Generalized Anxiety (*B*) | 0.800 [0.591, 1.009]*** | 1.081 [0.403, 1.759]** |
| Depression (*B*) | 0.865 [0.611, 1.119]*** | 1.246 [0.422, 2.070]** |
| Suicidal Ideation (*OR*) | 1.277 [1.170, 1.399]*** | 1.743 [1.313, 2.344]*** |
| Suicide Attempt (*OR*) | 1.247 [1.116, 1.399]*** | 1.711 [1.224, 2.408]** |

*Note.* *N* = 493. *B* = unstandardized regression coefficient; *OR* = odds ratio; CI = confidence interval; ACEs = adverse childhood experiences. Values are estimates with 95% CIs in brackets (t-based for the linear models, profile-likelihood for the logistic models). The two domain sub-scores were entered simultaneously in each model, following the classification in Cronholm et al. (2015): conventional/household adversities (items 1-16) and community/expanded adversities (items 17-20). Generalized anxiety and depression were modeled with linear regression; suicidal ideation and suicide attempt were modeled with binary logistic regression (reference category = no endorsement). All models adjust for age, years in the U.S., sex, region, educational attainment, college enrollment, employment, income, and immigrant generation. Reference categories are male (sex), Northeast (region), less than high school (educational attainment), currently enrolled in college (college enrollment), unemployed (employment), less than $10,000 (income), and 1st generation (immigrant generation). Table 6 reports item-level endorsement rates for the individual ACE items.

**p* < .05. ***p* < .01. ****p* < .001.

**Table S2**

ACEs × Immigrant Generation Interaction Models Predicting Mental Health Outcomes

Panel A. Interaction models

| Outcome | ACEs (Total) | Immigrant Status: 2nd Gen | ACEs × 2nd Gen | Test of interaction |
| --- | --- | --- | --- | --- |
| Generalized Anxiety (*B*) | 1.109 [0.744, 1.473]*** | 0.597 [-0.826, 2.020] | -0.355 [-0.774, 0.064] | *F*(1, 474) = 2.77, *p* = .097 |
| Depression (*B*) | 1.290 [0.847, 1.733]*** | 1.416 [-0.313, 3.144] | -0.489 [-0.998, 0.020] | *F*(1, 474) = 3.57, *p* = .060 |
| Suicidal Ideation (*OR*) | 1.766 [1.424, 2.294]*** | 2.945 [1.467, 6.373]** | 0.713 [0.543, 0.901]** | χ²(1) = 8.56, *p* = .003 |
| Suicide Attempt (*OR*) | 1.687 [1.329, 2.251]*** | 1.858 [0.730, 5.474] | 0.733 [0.540, 0.954]* | χ²(1) = 5.45, *p* = .020 |

Panel B. Generation-specific ACE slopes

| Outcome | 1st Generation | 2nd Generation |
| --- | --- | --- |
| Generalized Anxiety (*B*) | 1.109 [0.744, 1.473]*** | 0.754 [0.548, 0.959]*** |
| Depression (*B*) | 1.290 [0.847, 1.733]*** | 0.801 [0.551, 1.050]*** |
| Suicidal Ideation (*OR*) | 1.766 [1.424, 2.294]*** | 1.260 [1.159, 1.375]*** |
| Suicide Attempt (*OR*) | 1.687 [1.329, 2.251]*** | 1.237 [1.112, 1.379]*** |

*Note.* *N* = 493 (1st generation *n* = 114; 2nd generation *n* = 379). *B* = unstandardized regression coefficient; *OR* = odds ratio; CI = confidence interval; ACEs = adverse childhood experiences. Values are estimates with 95% CIs in brackets. Total ACE score is mean-centered, so the ACEs (Total) coefficient is the association for 1st-generation participants (the reference generation) and the ACEs × 2nd Gen coefficient is the difference in that association for 2nd-generation participants; for the logistic outcomes an interaction odds ratio below 1.00 indicates a weaker ACE association among 2nd-generation participants. Generalized anxiety and depression were modeled with linear regression; suicidal ideation and suicide attempt were modeled with binary logistic regression (reference category = no endorsement). All models adjust for age, years in the U.S., sex, region, educational attainment, college enrollment, employment, and income. Reference categories are male (sex), Northeast (region), less than high school (educational attainment), currently enrolled in college (college enrollment), unemployed (employment), less than $10,000 (income), and 1st generation (immigrant generation). The test of interaction compares the main-effects model with the interaction model (*F* test for linear outcomes; likelihood-ratio χ² for logistic outcomes). Panel B reports the generation-specific ACE slopes recovered from the same interaction models by changing the reference generation; these differ slightly from the stratified estimates in Tables 2-5 because the interaction models constrain covariate coefficients to be common across generations.

**p* < .05. ***p* < .01. ****p* < .001.

**Table S3**

Sensitivity Analyses for the Suicidality Outcomes

Panel A. Firth-penalized logistic regression predicting suicide attempt

| Predictor | Overall | 1st Generation | 2nd Generation |
| --- | --- | --- | --- |
| ACEs (Total) | 1.27 [1.17, 1.39]*** | 1.45 [1.19, 1.81]*** | 1.22 [1.10, 1.35]*** |
| Age | 0.94 [0.86, 1.02] | 1.04 [0.86, 1.26] | 0.90 [0.82, 0.99]* |
| Sex: Female | 1.51 [0.90, 2.53] | 1.59 [0.53, 5.03] | 1.42 [0.80, 2.55] |
| Immigrant Status: 2nd Gen | 1.00 [0.56, 1.86] | — | — |

Panel B. Suicidality models adjusted for concurrent depression and anxiety symptoms

| Predictor | Suicidal Ideation | Suicide Attempt |
| --- | --- | --- |
| ACEs (Total) | 1.216 [1.119, 1.326]*** | 1.211 [1.091, 1.347]*** |
| Depression (PHQ-9) | 1.068 [1.016, 1.123]* | 1.063 [0.999, 1.134] |
| Generalized Anxiety (GAD-7) | 1.075 [1.011, 1.143]* | 1.063 [0.983, 1.150] |

*Note.* *OR* = odds ratio; CI = confidence interval; ACEs = adverse childhood experiences; PHQ-9 = Patient Health Questionnaire-9; GAD-7 = Generalized Anxiety Disorder-7. Values are odds ratios with 95% CIs in brackets. Panel A reports Firth penalized logistic regression (Firth, 1993) predicting lifetime suicide attempt, fitted with a parsimonious a priori covariate set (ACEs, age, sex, and, in the overall model, immigrant generation) to address the sparse number of attempt events among 1st-generation participants; because region is not included, the analytic samples for Panel A are larger than in Tables 2-5 (overall *n* = 530; 1st generation *n* = 124; 2nd generation *n* = 406). The 1st-generation age effect that reached significance in Table 5 (*OR* = 1.491) was not retained under the penalized model (*p* = .70). Panel B reports standard logistic regression models for suicidal ideation and suicide attempt with concurrent depression and anxiety symptoms added as covariates; these models retain the full covariate set used in Tables 4 and 5 (*N* = 493) and profile-likelihood confidence intervals; Panel A reports penalized profile-likelihood intervals and should be read as testing whether ACE associations are independent of current distress rather than as a formal mediation analysis. Reference categories are male (sex), Northeast (region), less than high school (educational attainment), currently enrolled in college (college enrollment), unemployed (employment), less than $10,000 (income), and 1st generation (immigrant generation).

**p* < .05. ***p* < .01. ****p* < .001.
